# Transcriptomic analysis and experimental verification reveal the involvement of PI3K/AKT signaling pathway in high-altitude cognitive dysfunction

**DOI:** 10.3389/fphys.2026.1781613

**Published:** 2026-04-23

**Authors:** Yu Xin, Chenyu Yang, Gege Wang, Huiping Ma, Linlin Jing

**Affiliations:** 1 Department of Pharmacy, The First Affiliated Hospital of Xi’an Jiaotong University, Xi’an, Shaanxi, China; 2 Department of Pharmacy, The 940th Hospital of Joint Logistics Support force of People’s Liberation Army (PLA), Lanzhou, Gansu, China

**Keywords:** high-altitude cognitive dysfunction, inflammation, oxidative stress, PI3K/Akt signaling pathway, transcriptomic analysis

## Abstract

**Introduction:**

Cognitive impairment is a common symptom for these people entering high altitude. Unfortunately, the potential molecular mechanisms are not totally clear. This study aimed to identify the genes and signaling pathways associated with high-altitude cognitive dysfunction (HACD) in mice.

**Methods:**

Male C57BL/6 J mice were allocated into two groups: control group and hypobaric hypoxia (HH) group. The cognitive function was assessed using novel object recognition test and Morris water maze test. The histological analysis was performed using Hematoxylin-Eosin (HE) staining and Nissl staining. Evans blue (EB) assay was performed to evaluate the integrity of the blood-brain barrier (BBB). The gene levels in hippocampal tissue were assessed via RNA-Seq technique. Differentially expressed genes (DEGs) were identified using the DESeq2 R package, followed by functional and pathway enrichment analyses. The protein-protein interaction (PPI) network was established for screening hub genes, which were subsequently validated by qRT-PCR. The related proteins were detected by Western blot.

**Results:**

HH exposure led to pathological changes in hippocampal tissue, accompanied by increased oxidative stress, inflammatory response, and BBB disruption, and then induced impaired cognitive function in mice. In the HACD mice, 178 DEGs (70 upregulated and 108 downregulated genes) were found, in comparison to the control, and 8 hub genes were identified. GO and KEGG enrichment analysis demonstrated that PI3K/AKT signaling pathway is a significantly enriched pathway, suggesting its potential involvement in the pathogenesis of HACD. Then, we performed validation experiments via qRT-PCR for four hub genes (*Vwf*, *Vegfa*, *Kdr*, *Spp1*) closely related to the PI3K/AKT signaling pathway, and the results aligned with the RNA-seq data. Furthermore, Western blot analysis indicated that the PI3K/AKT pathway was substantially inhibited following HH exposure. Downstream analysis revealed significantly decreased expression of antioxidant proteins Nrf2 and HO-1, accompanied by increased phosphorylation of NF-κB, indicating enhanced neuroinflammation and impaired antioxidant defenses.

**Conclusions:**

Our results reveal a significant association between PI3K/AKT signaling pathway inhibition and HACD and offer potential therapeutic targets for developing novel treatment strategies for HACD.

## Introduction

The high-altitude (HA) environment, predominantly characterized by hypobaric hypoxia (HH), represents one of the most physiologically challenging conditions on Earth (Jiang et al., 2022). The reduced atmospheric pressure causes decreased oxygen partial pressure and blood oxygenation saturation (SpO_2_), triggering a range of physiological adaptations (Aboouf et al., 2023). As one of the highest oxygen‐consuming organs, the brain, especially the hippocampus, is particularly susceptible and vulnerable to reduced oxygen supply at HA, which can lead to cognitive impairment (Yang et al., 2023b). However, effective ways for the prevention and treatment of high-altitude cognitive dysfunction (HACD) are limited. One of the primary reasons for this limitation is that the pathophysiology of HACD remains incompletely understood. While several mechanisms have been reported, including the release of reactive oxygen species (ROS), inflammatory response, apoptosis, mitochondrial dysfunction, and enhanced blood-brain barrier (BBB) permeability (Chen et al., 2023; Zhang et al., 2024b), these findings fail to fully explain the complex processes underlying the onset and progression of HACD. Therefore, a deeper insight into the mechanisms driving HACD is essential for developing of more effective treatments.

Transcriptome analysis is an efficient sequencing technology that reveals specific biological processes and molecular mechanisms in the pathogenesis of diseases by screening and analyzing differentially expressed genes (DEGs) and their functions (Dong and Chen, 2013). RNA-sequencing (RNA-seq) combined with bioinformatics analysis has been widely employed in various areas of high altitude medicine, such as elucidating the mechanisms of high-altitude adaptation (Xin et al., 2020; Manella et al., 2022) and injury (Li et al., 2017; Han et al., 2024; Li et al., 2024a), and exploring potential therapeutic targets for drugs (Tian et al., 2024; Zeng et al., 2024). These efforts have yielded insights into new therapeutic opportunities.

The aims of this research were as follows: (1) to establish HACD mice model, which was evaluated by behavioral tests, pathological and biochemical alterations; (2) to determine the gene expression levels in the hippocampus of mice with or without HACD; (3) to assess the DEGs in response to HH stress, with a focus on identifying key pathways that drive HACD; (4) to validate the responsive genes and proteins associated with the key pathway. The results of this research may facilitate a deeper comprehension of the mechanisms involved in HACD and offer novel strategies to combat this disorder.

### Materials and methods

#### Reagents and antibodies

The kits for hydrogen peroxide (H_2_O_2_), Malondialdehyde (MDA), Superoxide Dismutase (SOD), and glutathione (GSH) were acquired from Nanjing Jiancheng Bioengineering Institute (Nanjing, China). ELISA kits for tumor necrosis factor-α (TNF-α), interleukin-1β (IL-1β), interleukin-6 (IL-6), and interleukin-10 (IL-10) were purchased from Wuhan Yangene Biological Technology Co., LTD (Wuhan, China). Evans Blue and formamide were purchased from Aladdin Chemical Reagent Co. Ltd. (Shanghai, China). The BCA protein assay kit was sourced from Beijing Solarbio Science & Technology Co., Ltd (Beijing, China). Primary antibodies against PI3K (ab183957) and p-PI3K (ab32089) were obtained from Abcam (Cambridge, UK). Primary antibodies against AKT (9272S) p-AKT (4060S), p-NF-κb (3033S), NF-κb (8242S), HO-1 (ab13243), ZO-1 (8193S), and occludin (91131S) were obtained from Cell Signaling Technology, Inc. (Danvers, USA). Primary antibodies against Nrf2 (16396-1-AP) was obtained from Proteintech (Wuhan, China). Primary antibody against *β*-actin were purchased from Zhongshan Golden Bridge Biological Technology Co., Ltd. (Beijing, China). HRP-conjugated Goat anti-mouse IgG and Goat anti-rabbit IgG were obtained from Proteintech (Wuhan, China).

### Animal model establishment

6–8 weeks-old Male C57BL/6 mice were obtained from the SPF (Beijing) biotechnology co., Ltd. (Beijing, China). The mice were raised in standard environment with a temperature of 23-25 °C and a 12-hour light-dark cycle and provided food and water *ad libitum*. The mice were randomly allocated to either a control group or a hypobaric hypoxia (HH) group, with 16 mice per group. After 7 days of adaptation, Morris water maze (MWM) train were conducted for five consecutive days. Then mice in HH group were exposed to an animal decompression chamber (DYC-3070, Guizhou Fenglei, China) simulating an altitude of 8–000 m (18% O_2_, 0.035 MPa, humidity, 40-50%, and temperature, 23-25 °C). These parameters were continuously monitored by the chamber’s integrated sensors and automatically recorded every 30 minutes to ensure stable exposure conditions throughout the experiment. The Con group mice were maintained under normal laboratory conditions (altitude, 1–400 m, humidity, 40-50%, temperature, 23-25 °C). After 72 h, behavioral tests were performed. Subsequently, the mice were deeply anesthetized with 5% isoflurane via inhalation, and then euthanized via cervical dislocation. The brain tissues were extracted, and the hippocampal regions were isolated and stored at -80 °C for further experiments. All animal procedures were carried out in accordance with ARRIVE guidelines and approved by the Biomedical Ethics Committee of the Health Science Center of Xi’an Jiaotong University (XJTUAE2025-415). A statement to confirm that all methods were carried out in accordance with relevant guidelines and regulations.

### Behavioral test

Novel object recognition (NOR) test was employed to assess episodic-like declarative memory of mice and included three periods (n=8 per group). The adaptation period was conducted 24 h after exposure to the HH chamber, allowing the mice to freely explore in a moving box without objects for a period of 5 min. The learning period was performed 48 h after exposure to the HH chamber, allowing the mice to freely explore in a moving box with two identical objects for 5 min. The test period was performed 72 h after HH exposure, allowing the mice to freely explore for 5 min in a box in which an old object was replaced with a new one. To eliminate any potential confounding factors, the moving box was wiped down with alcohol before and after the experiment to eliminate any extraneous odors. The speed, moving distance, and time taken to the new and old objects were collected. The recognition index (RI) was defined by the formula: RI = Time taken to the novel object/(Time taken to the both objects) × 100%.

Morris water maze (MWM) test was employed to assess the spatial memory of mice (n=8 per group). During the five consecutive days of the positioning navigation experiment, every mouse was released from four distinct quadrants and allowed to search for the fixed platform for up to 60 s. The latency, defined as the time taken for the mouse to locate the fixed platform, was recorded. Upon arrival at the platform, the mice were permitted to stay on the platform for 20 seconds and then placed back in the home cage. Following HH exposure, a space exploration experiment was administered to evaluate reference memory consolidation. Mice were placed from a randomly selected quadrant without platform, and their tracks were monitored for 60 seconds. Speed, entries (number of crossings of the platform), swimming distance and swimming time in the target quadrant, were recorded.

### Histological analysis

The pathological changes of hippocampal tissue in two groups of mice were detected. The collected brain tissues (including hippocampus, n=3 per group)) were fixed in 4% paraformaldehyde, embedded in paraffin and cut into 5 *μ*m thickness sections. These sections were used for Hematoxylin-Eosin (HE) staining and Nissl staining.

### Biochemical analysis

Hippocampal tissues (n=6 per group) were homogenized in saline to obtain homogenate, which was then centrifuged to get the supernatant. The levels of H_2_O_2_, MDA, SOD, and GSH in the supernatant were quantified utilizing corresponding kits (Nanjing Jiancheng Bioengineering Institute, Nanjing, China) as per the manufacturer’s guidelines. Protein concentrations were determined using a BCA protein assay kit, and the results were normalized to total protein content (expressed as per mg protein).

### ELISA assay

Hippocampal tissues (n=6 per group) were homogenized in PBS to obtain homogenate, which was then centrifuged to get the supernatant. The concentrations of TNF-α, IL-6, IL-1β, and IL-10 were measured utilizing corresponding ELISA kits (Wuhan Yangene Biological Technology Co., LTD, Wuhan, China) as per the manufacturer’s manual. Protein concentrations were determined using a BCA protein assay kit, and the results were normalized to total protein content (expressed as per mg protein).

### Evans blue assay

After HH exposure, mice were immediately injected with 2% EB solution (100 µL/20 g body weight) via the tail vein. One hour post-injection, the animals were euthanized and perfused with saline, after which the whole brains were harvested and the hippocampus were dissected. The isolated hippocampal tissues (n=6 per group) were precisely weighed and homogenized in formamide at a ratio of 1 mL per 0.1 g of tissue. The homogenate was incubated overnight at 60 °C and then centrifuged at 16,000 rpm for 30 minutes. The supernatant was carefully collected, transferred to a 96-well plate (200 µL per well), and the absorbance was measured at 620 nm using a microplate reader. Evans blue content was quantified using a standard curve generated by serial dilution of EB standard solution (concentration gradient: 10, 5, 2.5, 1.25, 0.625, and 0 µg/mL). The final concentration of EB in the samples was expressed as µg/g tissue.

### Total RNA extraction and transcriptome sequencing analysis

RNA-seq analysis was conducted by Novogene Co., Ltd (Beijing, China). In brief, total RNA was extracted from hippocampal tissues (n=4 per group) utilizing the TRIzol reagent kit (Invitrogen, CA, USA) as per the manufacturer’s guidelines. A total of eight RNA samples were prepared, with four replicates per group. RNA quantification and purity were measured using a NanoDrop 2000 Spectrophotometer (Thermo Scientific, USA), while RNA integrity was verified utilizing the Agilent 5400 Bioanalyzer (Agilent Technologies, Santa Clara, CA, USA). The libraries were established with Fast RNA-seq Lib Prep Kit V2 (Cat. No. RK20306) and sequenced on an illumina Novaseq 6000 platform, producing 150 bp reads. The fastp software was employed to remove low-quality sequences from the raw data and retain clean reads for further analysis. Clean reads were matched to the mouse reference genome (mm10) using HISAT2, and gene expression levels were quantified using featureCounts v1.5.0-p3. FPKM values were calculated to estimate gene expression abundance. Principal Component Analysis (PCA) analysis was conducted using R (v 3.5.0). DEGs were identified using the DESeq2, with a significance threshold set at p_adj_ value ≤ 0.05 and |log_2_FoldChange| ≥ 1. To visualize the DEGs, the R packages “ggplot2” (version 3.3.6) and “pheatmap” (version 1.0.12) were used to draw volcano maps and heat maps, respectively.

### Protein-protein interaction network construction of core targets and identification of hub genes

The PPI network of overlapping targets was constructed using the STRING database (https://cn.string-db.org/). The analysis was restricted to “Mus musculus” with a medium confidence interaction score threshold (> 0.4) to ensure reliable identification of functionally relevant protein interactions corresponding to the target genes. The protein interaction data obtained from STRING were imported into Cytoscape for visualization and topological analysis. Using the cytoHubba plugin, we calculated the “Nodes Score” via five algorithms: MCC (Maximum Clique Centrality), MNC (Neighborhood Component Centrality), Degree Centrality, Closeness Centrality, and Radiality Centrality, selecting the top 10 ranked genes from each as key candidates. Hub genes were identified as the overlapping targets of the five algorithms.

### Gene function and pathway enrichment analysis

Enrichment analysis of DEGs, including Gene Ontology (GO) and Kyoto Encyclopedia of Genes and Genomes (KEGG), was performed utilizing the clusterProfiler package in R (v 3.5.0). The GO enrichment analysis systematically evaluated three functional categories: Biological processes (BP)-identifying key physiological activities; Cellular components (CC)-determining subcellular localization; Molecular functions (MF)-characterizing biochemical activities. KEGG pathway analysis was conducted to uncover significant signaling pathways associated with the candidate targets. Statistical significance thresholds were maintained as previously described, where p-value below 0.05 was considered significant enrichment.

### Quantitative real-time PCR analysis

Total RNA was isolated from hippocampal tissue (n=6 per group) and reverse transcribed into cDNA using FastKing gDNA Dispelling RT SuperMix (KR118, Tiangen, China). qRT-PCR was carried out using SYBR Green (FR227, Tiangen, China) on a ViiA7 system (Applied Biosystems, USA) as per the manufacturer’s guidelines. *β*-actin served as an internal normalizer. The relative level of gene was quantified using the 2^-ΔΔCT^ method. Primers were designed and synthesized by Wuhan Servicebio Technology Co., Ltd. The sequences are provided in **Table 1**.

**Table 1:** Primer sequences for the genes observed in qRT-PCR.

Gene	Forward Primer (5'-3')	Reverse Primer (5'-3')
*β*-actin	GTGACGTTGACATCCGTAAAGA	GTAACAGTCCGCCTAGAAGCAC
Kdr	TTTGGCAAATACAACCCTTCAGA	GCAGAAGATACTGTCACCACC
Spp1	TTTCACTCCAATCGTCCCTACA	CTGCCCTTTCCGTTGTTGTC
Vwf	GCCCAGGAAGCTATCAGCC	ATACACGAAGCCACTCTCGTC
Vegfa	TAACGATGAAGCCCTGGAGTG	CACAGTGAACGCTECAGGATTTA

### Western blot analysis

Protein from hippocampal tissue (n=3 per group) was sufficiently lysed with RIPA buffer supplemented with protease and phosphatase inhibitor and separated by SDS-PAGE. Subsequently, the proteins were transferred onto PVDF membranes. After being blocked in 5% nonfat milk in TBST, the membranes were incubated with primary antibodies overnight at 4 °C and then incubated with the secondary antibody for 1 h at RT. Protein bands were visualized using a chemiluminescence instrument (ChemiDoc MP Imaging System, Bio-Rad), and then analyzed using ImageJ software.

### Statistical analysis

All data were presented as mean ± standard deviation (SD). GraphPad Prism version 8 (La Jolla, CA, USA) was employed for statistical analyses. Data from the positioning navigation experiment were subjected to two-way repeated measures ANOVA, with treatment (Con vs. HH) and training day (days 1–5) as between- and within-subjects factors, respectively. For other date, Student’s t test was conducted for two groups comparisons. *p* values less than 0.05 were considered statistically significant differences. Spearman’s correlation coefficient was employed to evaluate the correlation between samples.

## Results

### HH induced cognitive impairments

**Figure 1:**
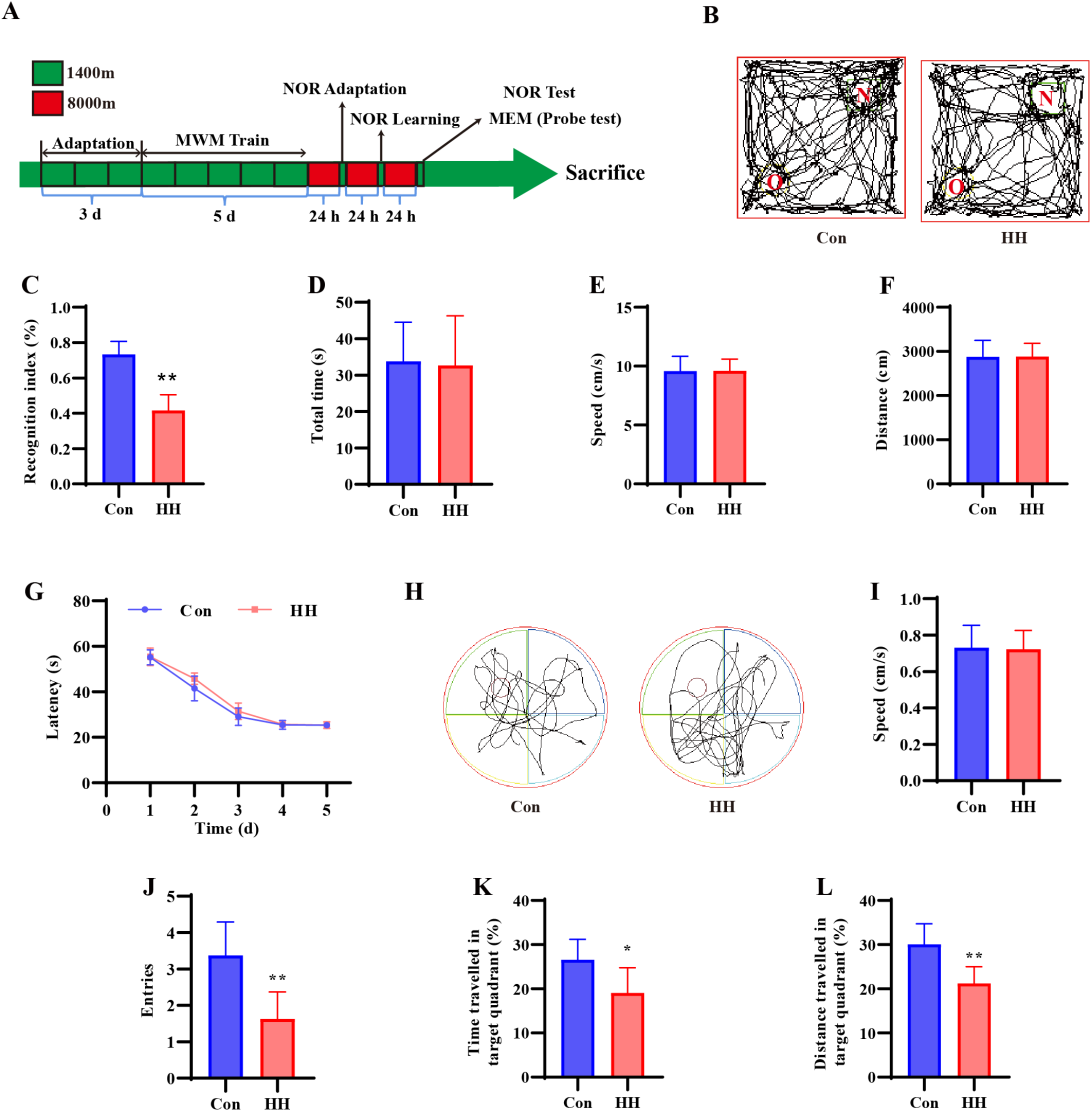
HH induced cognitive dysfunction in mice. **(A)** Experimental design diagram. NOR experimental results: **(B)** Representative images of the NOR test. **(C)** Recognition index. **(D)** Total time. **(E)** Moving distance. **(F)** Speed. MWM experimental results: **(G)** Latency (Time to find the platform during the positioning navigation experiment). **(H)** Representative images of the MWM test. **(I)** Average swimming speed. **(J)** Frequency of crossing the target quadrant. **(K)** Proportion of swim time (swimming time in the target quadrant/total swimming time × 100%). **(L)** Proportion of swim distance (swimming distance in the target quadrant/total swimming distance × 100%). Data are presented as mean ± SD. Statistical comparisons were performed by Student’s t test. n = 8 per group, ^*^p < 0.05, ^**^p < 0.01 vs. Con group.

To explore the impact of HH on cognitive dysfunction, NOR test and MWM test were conducted. The experimental timeline is depicted in **Figure 1A**.

In the NOR test, which reflects the episodic-like declarative memory of mice by updating objects (**Figure 1B**), HH group mice showed a lower RI than that of Con mice (*p* < 0.01, **Figure 1C**). No significant differences were found in total time (**Figure 1D**), speed (**Figure 1E**) and moving distance (**Figure 1F**) between two groups, indicating that HH specifically impairs memory without affecting motor function. These findings demonstrated that HH exposure negatively impacts memory in mice.

The MWM test, a widely used experiment for evaluating spatial memory, revealed that both groups of mice showed a gradual reduction in escape latency over the 5-day training period (**Figure 1G**), indicating the formation of short-term spatial memory. During the space exploration experiment (**Figure 1H**), no significant differences were found in swimming speed between two groups (**Figure 1I**), confirming that motor abilities remained unaffected by HH. However, mice in the HH group exhibited a remarkable decrease in the number of enters into the targeted quadrant (*p* < 0.05, **Figure 1J**), percentages of swimming time (*p* < 0.05, **Figure 1K**) and swimming distance (*p* < 0.05, **Figure 1L**) within the targeted quadrant, compared to the Con group.

### HH induced pathological changes in mouse brain tissue

**Figure 2:**
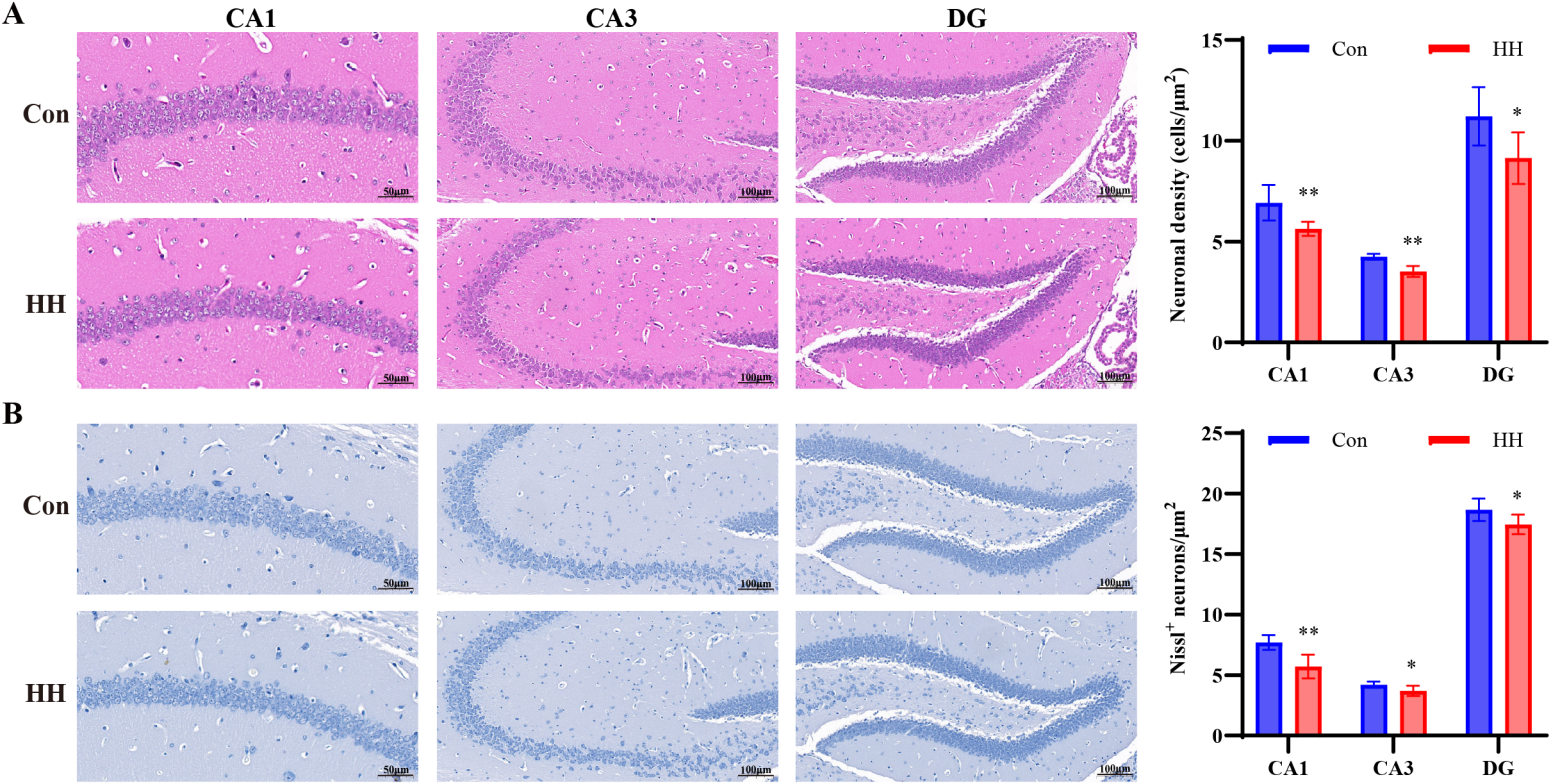
HH induced hippocampal tissue pathological changes. **(A)** H&E staining and quantification of neuronal density in the CA1, CA3, and DG regions of the hippocampus. **(B)** Nissl staining and quantification of Nissl^+^ cell counts in the CA1, CA3, and DG regions of the hippocampus. Data are expressed as mean ± SD. Statistical comparisons were performed by Student’s t test. n = 3 mice per group, ^*^p < 0.05, ^**^p < 0.01 vs. Con group.

Histopathological examination of brain tissues using H&E staining revealed distinct morphological alterations between the Con and HH groups. In the Con group, hippocampal neurons showed normal morphology, with regular arrangement and uniform distribution. In contrast, the HH group displayed disorganized pyramidal cell layers and a noticeable reduction in neuronal density in the hippocampal CA1, CA3, and DG regions (*p* < 0.01 or *p* < 0.05, **Figure 2A**). These observations were further corroborated by Nissl staining, which exhibited a significant decrease of Nissl-positive neurons in the hippocampal CA1, CA3, and DG regions of HH-exposed mice (*p* < 0.01 or *p* < 0.05, **Figure 2B**). These findings indicate that HH exposure disrupts neuronal architecture and function in the hippocampus, potentially contributing to cognitive impairments.

### HH induced oxidative stress in hippocampal tissue

**Figure 3:**
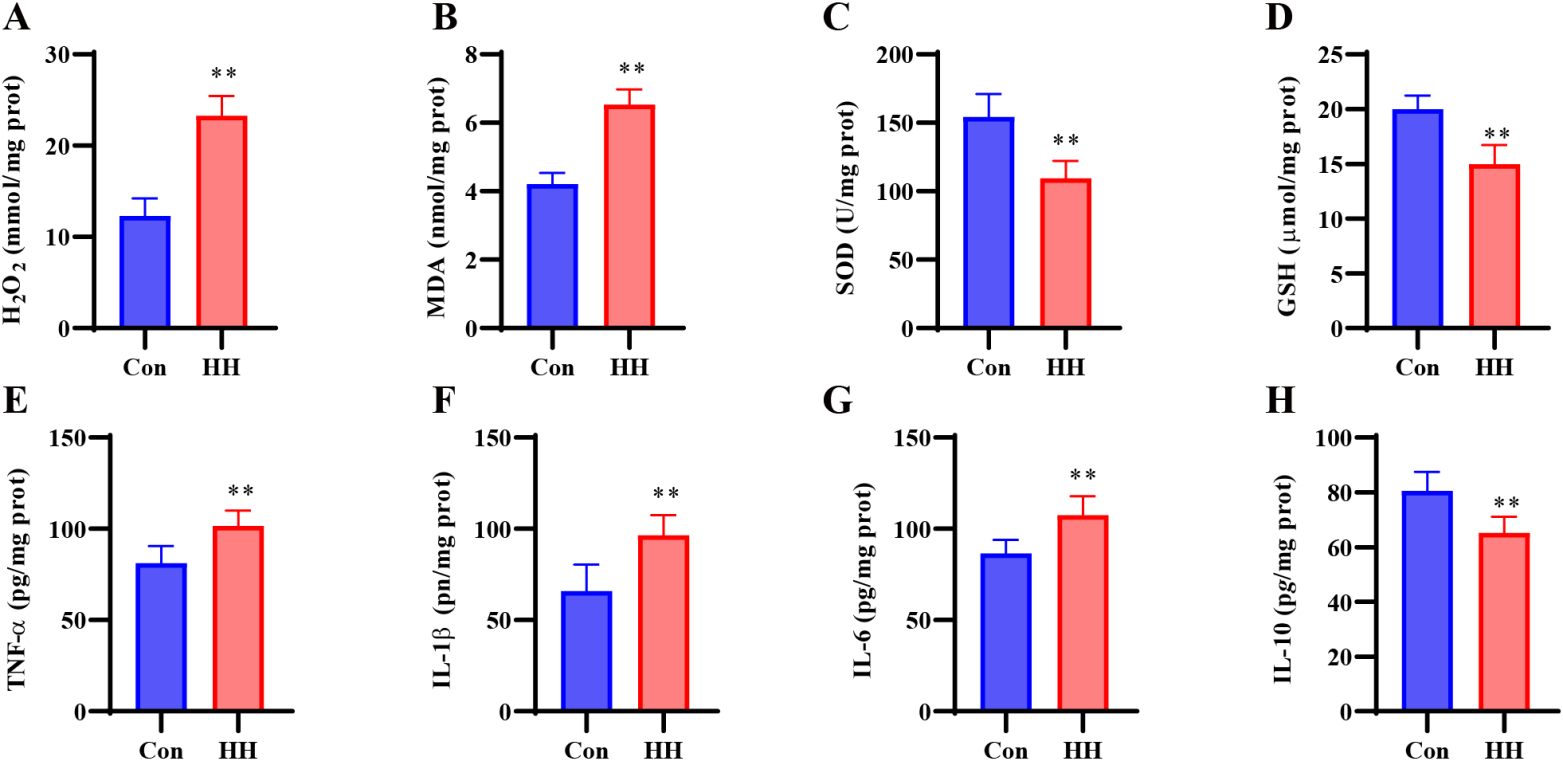
HH induced oxidative stress and inflammatory response in the hippocampal of mice. **(A)** H_2_O_2_ level. **(B)** MDA level. **(C)** SOD activity. **(D)** GSH level. **(E)** TNF-α level. **(F)** IL-6 level. **(G)** IL-1β level. **(H)** IL-10 level. Data are expressed as mean ± SD. Statistical comparisons were performed by Student’s t test. n = 6 per group, ^**^p < 0.01 vs. Con group.

As seen in **Figures 3A–D**, the contents of H_2_O_2_ and MDA were remarkably elevated, while SOD activity and GSH content were remarkably decreased in the hippocampal tissue following HH exposure (*p* < 0.01). These data demonstrate that HH stimulation led to oxidative stress in the hippocampal tissue.

### HH induced inflammatory response in hippocampal tissue

As seen in **Figures 3E–H**, concentrations of pro-inflammatory cytokines (TNF-α, IL-6, and IL-1β) were remarkably elevated (*p* < 0.01), while the content of anti-inflammatory cytokine IL-10 was remarkably decreased in the hippocampal tissue following HH exposure (*p* < 0.01). These findings demonstrate that HH stimulation led to inflammatory response in the hippocampus.

### HH induced BBB dysfunction

**Figure 4:**
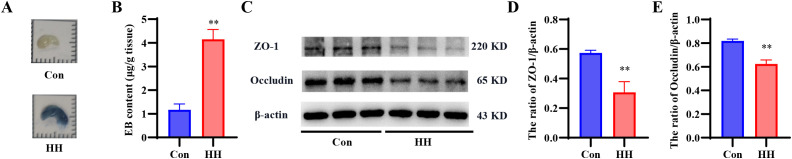
Effects of HH on the BBB integrity in mouse hippocampus. **(A)** Representative images of EB extravasation in the hippocampus. **(B)** Quantitative analysis of EB leakage. **(C)** Representative western blots of ZO-1 and occludin. Quantitative analysis of the ZO-1/β-actin **(D)** and occludin/β-actin **(E)** ratios. Data are presented as mean ± SD. Statistical comparisons were performed by Student’s t test. For EB, n = 6 per group; for Western blot analysis, n = 3 per group, ^**^p < 0.01 vs. Con group.

EB extravasation was first assessed to evaluate BBB permeability in the hippocampus (**Figures 4A, B**). As expected, the BBB of control mice remained intact, as evidenced by the absence of EB leakage. In contrast, exposure to HH led to marked EB extravasation and accumulation in the hippocampal tissue (*p* < 0.01). We then examined the expression of tight junction proteins, ZO-1 and Occludin, via Western blot (**Supplementary Figure S1**). Consistent with the increased BBB permeability, HH exposure resulted in significantly decreased expression of both ZO-1 and Occludin compared to the control group (*p* < 0.01, **Figures 4C–E**), indicating compromised structural integrity of the BBB.

### Transcriptome profiling of hippocampal tissue

**Figure 5:**
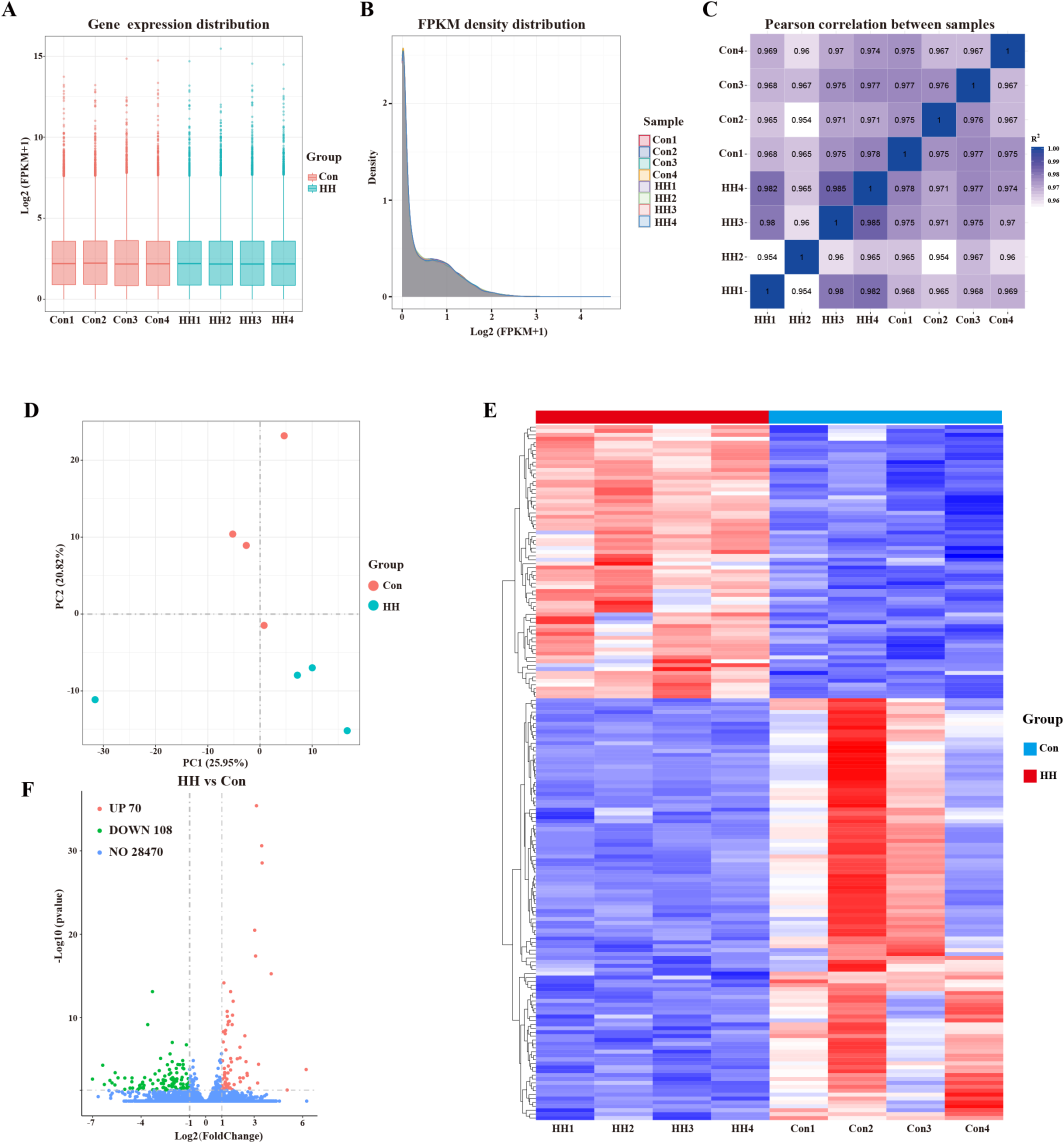
Features of RNA−sequencing data. **(A)** Boxplots of log2 (RPKM + 1) expression values for each sample. **(B)** Density plot of the distribution of log2 (RPKM + 1) values for each sample. **(C)** Pearson’s correlation analysis between samples. **(D)** PCA analysis. **(E)** Heatmap analysis of DEGs between the Con and HH groups. **(F)** Volcano plot analysis of DEGs between the Con and HH groups.

The quality of the RNA sequencing data was evaluated, with Q30 base distributions ranging from 93.33% to 93.92% (**Supplementary Table S1**), confirming high data reliability for downstream analysis. Clean reads were aligned to the mouse reference genome, achieving mapping ratios between 92.36% and 95.52% (**Supplementary Table S2**). The distribution of gene expression across all eight datasets is illustrated in boxplots (**Figure 5A**), while density plots revealed similar distribution characteristics on chromosomes between the Con and HH groups (**Figure 5B**). High reproducibility of the sequencing results was demonstrated by Pearson’s correlation coefficients exceeding 0.99 for matched biological replicates. Within-group correlations were also strong, with coefficients greater than 0.95. (**Figure 5C**). PCA further confirmed clear separation between the Con and HH groups in the PC1×PC2 score plot (**Figure 5D**). To identify differentially expressed genes (DEGs) under HH conditions, we conducted comparative analyses between the two groups, visualized through cluster heatmaps (**Figure 5E**) and volcano plots (**Figure 5F**). Compared to the Con group, 178 DEGs (70 upregulated and 108 downregulated) were obtained in the hippocampal tissue of mice in the HH group.

### Bioinformatics analysis

**Figure 6:**
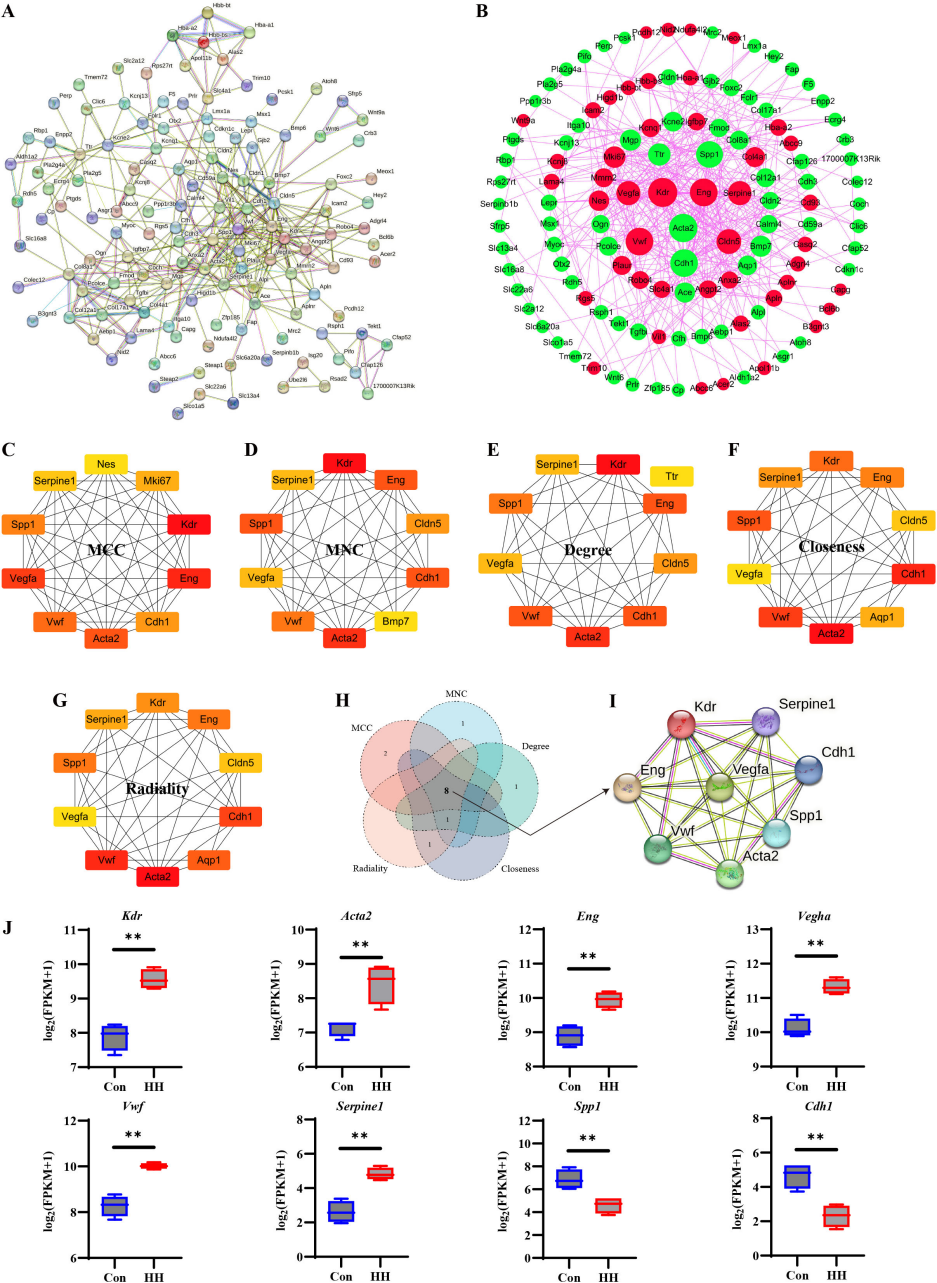
Screen of the hub targets from the DEGs. **(A)** PPI network by stinging. **(B)** PPI network by Cytoscape. Red circles indicate significantly upregulated DEGs, while green circles indicate significantly downregulated DEGs. Node size corresponds to the degree value. **(C)** Top 10 hub genes determined by the MCC algorithm. **(D)** Top 10 hub genes determined by the MNC algorithm. **(E)** Top 10 hub genes determined by the Degree algorithm. **(F)** Top 10 hub genes determined by the Closeness algorithm. **(G)** Top 10 hub genes determined by the Radiality algorithm. **(H)** Venn diagram for screening overlapping hub genes derived from five algorithms. **(I)** PPI network of the 8 core genes derived from STRING database. **(J)** Relative levels of 8 core genes between two groups. ^**^p < 0.01.

178 DEGs were subjected to PPI analysis using the STRING database (**Figure 6A**) and then visualized in Cytoscape software, generating a network with 123 nodes and 367 edges, excluding unconnected proteins (**Figure 6B**). Red indicates upregulated genes, and green indicates downregulated genes. These targets were ranked by their “Degree value”, where larger circle sizes correspond to stronger interactions. To comprehensively characterize the core genes mediating the development of HACD, we employed five distinct topological analysis methods available in the CytoHubba plugin. For each algorithmic approach, the top 10 genes were selected as the hub genes (**Figures 6C–G**). Subsequent the core genes identified by the five algorithms were intersected (**Figure 6H**), and a total of eight genes were identified, namely kinase insert domain receptor (*Kdr*), von Willebrand factor (*Vwf*), secreted phosphoprotein 1 (*Spp1*), endoglin (*Eng*), actin alpha 2, smooth muscle (*Acta2*), cadherin 1 (*Cdh1*), serpin family E member 1 (*Serpine1*), and vascular endothelial growth factor A (*Vegfa*), which were defined as the core gene in the development of HACD. A targeted PPI network was generated to map interactions among these core genes (**Figure 6I**). Compared to the Con group, HH exposure significantly decreased the levels of *Spp1* and *Cdh1* (*p* < 0.01), while significantly increasing the levels of *Kdr*, *Eng*, *Vegfa*, *Acta2*, *Vwf*, and *Serpine1* (*p* < 0.01, **Figure 6J**).

**Figure 7:**
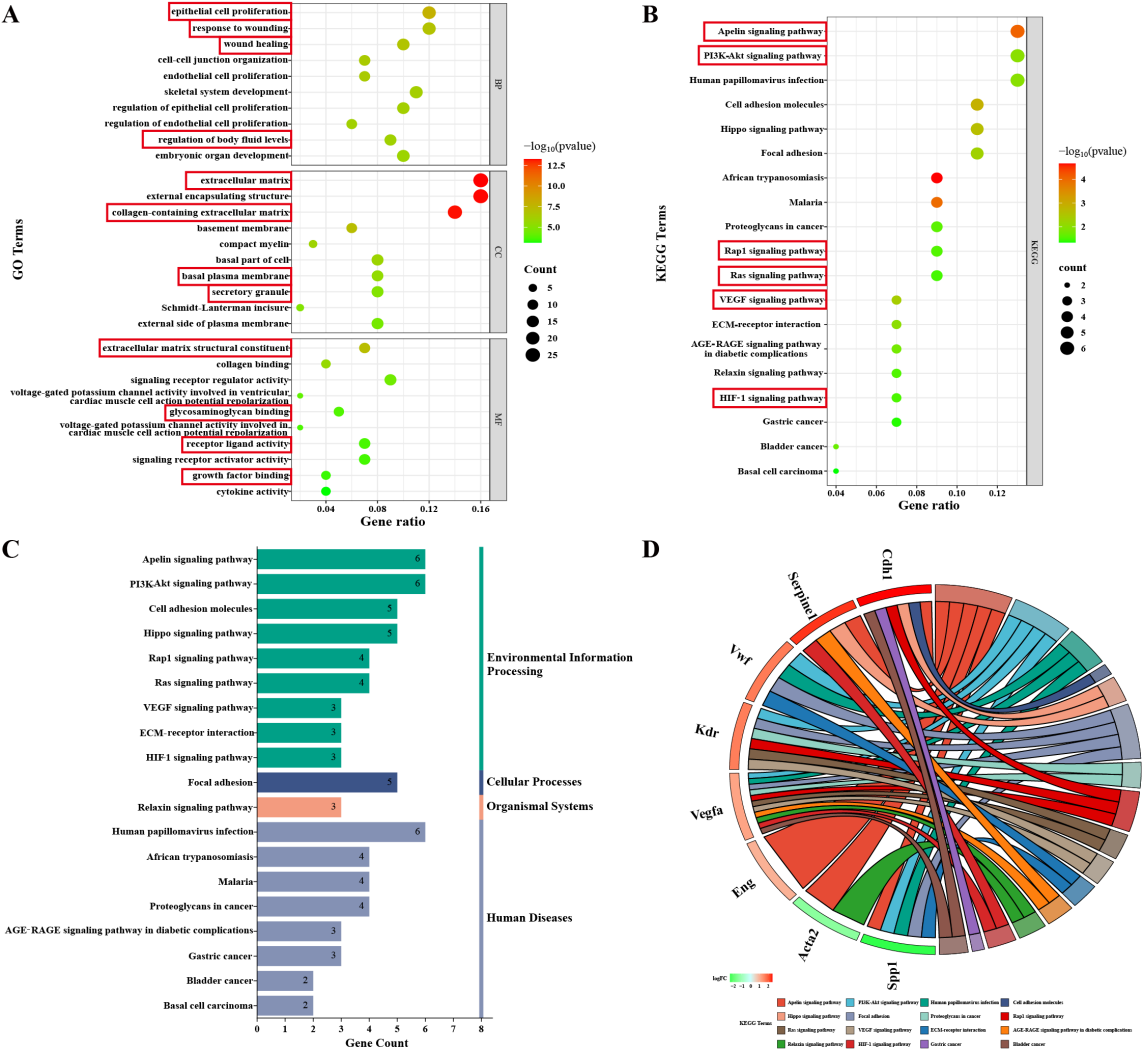
Bioinformatic analysis. **(A)** GO analysis of DEGs in the Con and HH groups, showing the top 10 categories with the least p values for each classification. **(B)** KEGG analysis of DEGs in the Con and HH groups, highlighting the significantly enriched pathways ordered by p values. **(C)** Categorical histograms of the significantly enriched pathways. **(D)** Chord plot illustrates the detailed relationship between core genes and the significantly enriched pathways.

178 DEGs were then functionally annotated using Gene Ontology (GO) analysis, by which identified 843 significantly enriched terms, comprising 693 terms in BP, 43 terms in CC, and 107 terms in MF. The top 10 enriched GO terms from each ontology (BP, CC, MF) ranked by p value were identified and graphically represented in a bubble chart (**Figure 7A**). BP analysis indicated that DEGs were primarily enriched in response to wounding, wound healing, endothelial cell proliferation, and regulation of body fluid levels. For CC, DEGs were primarily located in extracellular matrix, collagen-containing extracellular matrix, basal plasma membrane, and secretory granule. For MF, DEGs were mainly enriched in extracellular matrix structural constituent, glycosaminoglycan binding, receptor ligand activity, and growth factor binding.

KEGG pathway enrichment analysis revealed 19 significantly enriched signaling pathways, which were visualized in a KEGG bubble chart (**Figure 7B**) and further categorized in a histogram (**Figure 7C**). Among these pathways, several relevant signaling cascades were identified, including Apelin signaling pathway, PI3K-Akt signaling pathway, Rap1 signaling pathway, Ras signaling pathway, VEGF signaling pathway, and HIF-1 signaling pathway. Detailed relationship between core genes and these significantly enriched pathways were presented by chord plot (**Figure 7D**). Four core genes, including *Kdr*, *Vegfa*, *Vwf*, and *Spp1*, participated in the PI3K-Akt signaling pathway. These findings underscore the critical role of the PI3K/AKT signaling pathway in the development of HACD.

### Validation of RNA-seq data using qRT-PCR

**Figure 8:**
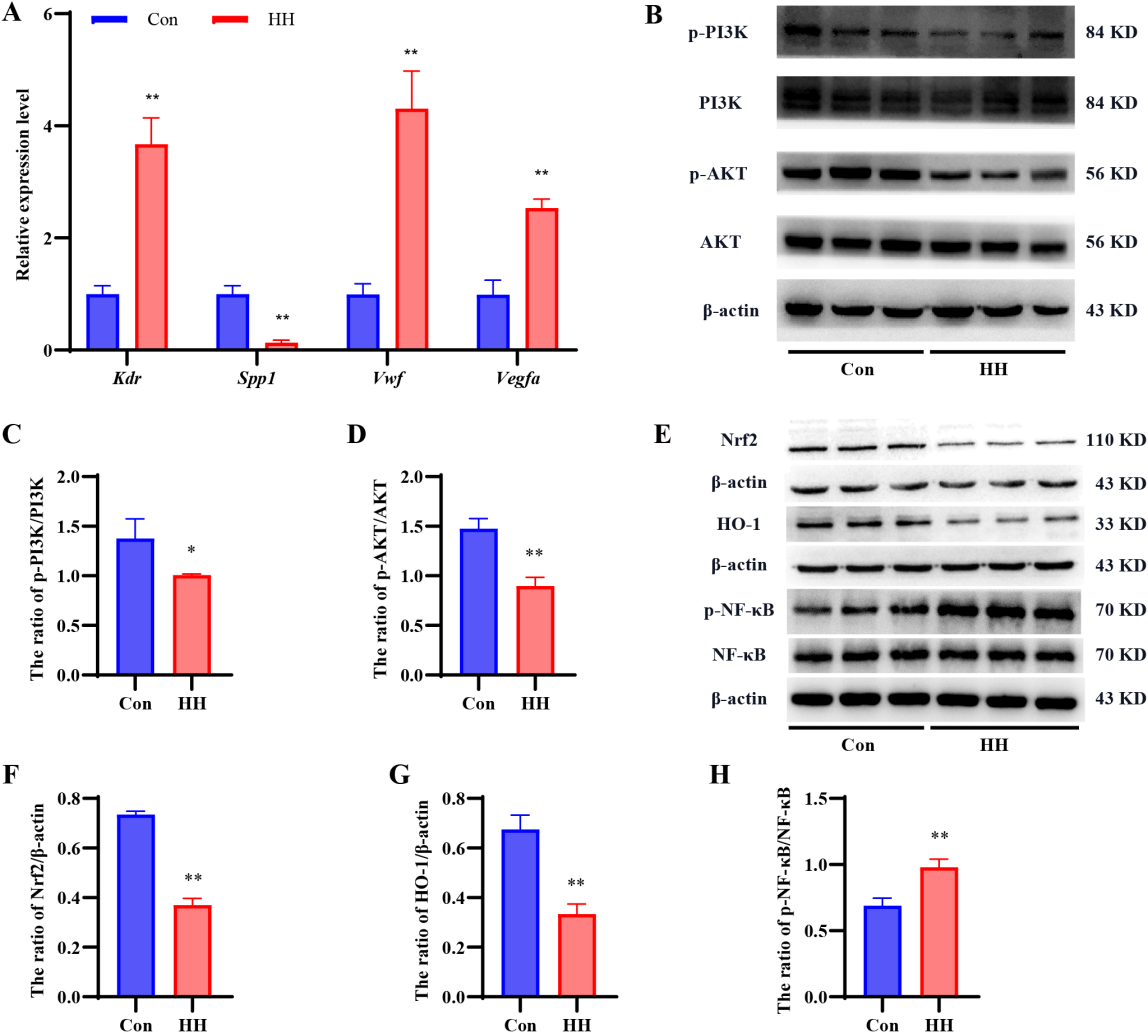
Effects of HH on the expression of genes and proteins related to PI3K/AKT signaling pathway in mouse hippocampus. **(A)** The relative mRNA expression levels of *Kdr*, *Spp1*, *Vwf*, and *Vegfa* screened via qRT-PCR. **(B)** Representative western blots of p-PI3K, PI3K, p-AKT, and AKT. Quantitative analysis of the p-PI3K/PI3K **(C)** and p-AKT/AKT **(D)** ratios. **(E)** Representative western blots of Nrf2, HO-1, p-NF-κB, and NF-κB. Quantitative analysis of the Nrf2/β-actin **(F)**, HO-1/β-actin **(G)**, and p-NF-κB/NF-κB **(H)** ratios. Data are presented as mean ± SD. Statistical comparisons were performed by Student’s t test. For qRT-PCR, n = 6 per group; for Western blot analysis, n = 3 per group, ^*^p < 0.05, ^**^p < 0.01 vs. Con group.

To verify the reliability of the transcriptome sequencing results in our study, four core DEGs (1 downregulated and 3 upregulated) related to the PI3K/AKT signaling pathway screened by PPI were further quantified via qRT-qPCR. As illustrated in **Figure 8A**, HH exposure significantly decreased the levels of *Spp1* (*p* < 0.01), while markedly elevating the levels of *Vwf*, *Vegfa*, and *Kdr* (*p* < 0.01) compared to the Con group. These results corroborate the RNA-seq data, confirming its accuracy and reliability.

### HH induced cognitive impairment via regulating the PI3K/AKT signaling pathway

The RNA-seq analysis showed that the PI3K/AKT signaling pathway was associated with HACD. To further confirm this finding, the expression of key proteins in this pathway, including PI3K, p-PI3K, AKT, and p-AKT, was detected via Western blot (**Supplementary Figure S2**). As shown in **Figures 8B–D**, HH exposure significantly lowered the p-PI3K/PI3K and p-AKT/AKT ratios in hippocampal tissue (*p* < 0.01), compared to Con group, indicating that HH-induced cognitive dysfunction was closely associated with inhibition of the PI3K/AKT signaling pathway.

To further elucidate the downstream consequences of PI3K/AKT pathway inhibition, we examined the expression of Nrf2 and HO-1, two antioxidant proteins regulated by this pathway, as well as the phosphorylation level of NF-κB, a key inflammatory factor negatively modulated by PI3K/AKT signaling via Western blot (**Supplementary Figure S3**). As presented in **Figures 8E–H**, HH exposure significantly decreased the protein expression of Nrf2 and HO-1 in the hippocampal tissue (*p* < 0.01). Moreover, the ratio of phosphorylated NF-κB (p-NF-κB) to total NF-κB was markedly increased (*p* < 0.01), indicating NF-κB pathway activation. These results suggest that HH-induced inhibition of the PI3K/AKT pathway leads to suppression of the antioxidant defense system and concurrent activation of neuroinflammatory responses, both of which may be linked to the observed cognitive impairment.

## Discussion

HH, a primary physiological challenge in high-altitude environments, is known to induce cognitive dysfunction. However, the underlying mechanisms remain complex and not fully understood. Preventing HACD is a significant global challenge. Although many agents have shown efficacy in HACD, few have been used in clinical practice. Therefore, developing safe and effective drugs or strategies to prevent and treat HACD remains a critical research priority. In this study, we examined the characteristics of HACD and further explored potential mechanism through transcriptomic analysis. Our research provided direct evidence that oxidative stress and inflammation, regulated by the PI3K/AKT signaling pathway, are involved in HACD, offering potential new therapeutic targets.

Previous research indicated that even brief exposure to high altitudes (e.g., 15 minutes at 6096 m) can rapidly impair learning and memory functions, particularly memory encoding, retrieval, and retention (Nation et al., 2017). Our data also revealed that episodic-like declarative memory and learning and memory functions were impaired in mice exposed to HH, as demonstrated by a decreased DI in the NOR test and increased escape latencies in the MWM test. The hippocampus, a key brain region that controls learning and memory, is particularly susceptible to hypoxia (Eichenbaum, 2000). HH exposure can lead to neuronal fixation, degeneration, and apoptosis in hippocampal pyramidal neurons, which are likely key contributors to memory impairment (Maiti et al., 2007; Zhang et al., 2024a). Our histopathological analyses using HE and Nissl staining further confirmed HH-induced damage in the hippocampal region, aligning with prior studies.

Exposure to high altitude results in cognitive deficit accompanied by oxidative stress in the hippocampus (Lin et al., 2012). Oxygen is essential for cellular homeostasis and adenosine triphosphate (ATP) production via oxidative phosphorylation in the mitochondrial respiratory chain (Gnaiger, 2001). Reduced oxygen levels disrupt oxidative phosphorylation, resulting in the overgeneration of ROS (Kalyanaraman, 2013). Excessive ROS can destroy the basement membrane of the BBB, causing vasogenic edema, and disrupting the brain’s oxidative and antioxidant systems, ultimately resulting in cognitive impairment. In our study, HH exposure significantly increased levels of oxidative stress markers (H_2_O_2_ and MDA) while reducing antioxidant defenses (SOD and GSH) in the hippocampus, further supporting the role of oxidative stress in HACD.

HH exposure also triggers a robust inflammatory response. Pro-inflammatory cytokines such as IL-1β, IL-6, and TNF-α are significantly elevated under HH conditions (Pham et al., 2021). The hippocampus, which expresses abundant receptors for these cytokines, such as IL-1β and TNF-α receptors, is particularly susceptible to neuroinflammation (Rothwell et al., 1996). Thus, neuroinflammation-induced impairments in hippocampal structure and function contribute to diminished memory performance. Many studies have proven that excessive inflammation contributes, at least partially, to HACD (Li et al., 2024b). Our findings align with these observations, as the contents of pro-inflammatory cytokines significantly enhanced, while anti-inflammatory cytokines significantly reduced in the hippocampus of mice with HACD.

Following these tests, we conducted RNA-seq and transcriptome analysis of hippocampal tissue. HH exposure leads to changes in the expression of corresponding genes (Manella et al., 2022). Relying on transcriptomic methods to analyze the changes of related genes can further reveal the occurrence and mechanism underlying HACD and provide effective measures for prevention and treatment. In the current study, 695 DEGs (265 upregulated and 430 downregulated) between the Con and HH groups were obtained.

A key finding from our study is that 8 crucial targets related to HACD, including *Kdr*, *Eng*, *Vegfa*, *Acta2*, *Vwf*, *Spp1*, *Cdh1*, and *Serpine1*, were identified via PPI analysis. Notably, these core targets are closely associated with BBB homeostasis and are involved in regulating the structural integrity and physiological function of the BBB. Spp1 is responsible for encoding osteopontin (OPN), a neuroprotective glycoprotein (Meller et al., 2005) abundant in acidic phosphate (Wen et al., 2024). OPN treatment has been shown to inhibit the BBB disruption after subarachnoid hemorrhage (SAH) (Suzuki et al., 2010) and activated the PI3K/AKT signaling pathway (Pang et al., 2021). CDH1, also known as E-cadherin, is a calcium-dependent adhesion glycoprotein essential for maintaining tissue integrity and homeostasis (Zhang et al., 2025). As a key component of adherens junctions, it directly regulates cell adhesion and helps maintain tight junction structure across the brain endothelium (He et al., 2025). In our study, HH exposure downregulated the expressions of *Spp1* and *CDH1*, implicating that PI3K/AKT signaling pathway inhibition and BBB disruption are involved in HACD. Vegfa, which belongs to the VEGF family, is a permeability factor for promoting endothelial cell growth and angiogenesis. In addition, VEGF is reported to disrupt the barrier function of the BBB and increase its permeability by inducing tight junction protein degradation (Hu et al., 2022; Yang et al., 2024). Recent studies have identified that HH upregulated VEGF expressions (Chang et al., 2023). The *Kdr* gene encodes vascular endothelial growth factor receptor 2 (VEGFR-2), a transmembrane glycoprotein composed of 1,356 amino acids that is predominantly expressed in neurons and vascular endothelial cells (Shah et al., 2025). Overexpression of VEGFA activates downstream signaling pathways by binding to VEGFR2, leading to increased BBB permeability (Peng et al., 2024). VWF is an ultra-large multimeric glycoprotein that is only synthesized and stored in Weibel-Palade bodies (WPB) of endothelial cells (Zanetta et al., 2000). Previous studies have shown that raised Vwf exacerbates brain inflammatory responses, BBB disruption, and edema formation (Suidan et al., 2010; Zhu et al., 2016). Acta2, also known as alpha smooth muscle actin (α-SMA), is a major contractile protein expressed in brain vessels, thereby playing a crucial role in regulating blood vessel contraction (Yuan, 2015). Endoglin (ENG), a homodimeric transmembrane glycoprotein abundantly expressed on vascular endothelial cells including the brain endothelium (Matsubara et al., 2000), functions as an accessory TGF-β receptor that regulates endothelial cell proliferation and migration during angiogenesis (Tian et al., 2010). Notably, upon hypoxia, ENG is extensively upregulated and acts as a direct inflammatory mediator at the brain endothelium (Haarmann et al., 2022). Serpine1, also known as plasminogen activator inhibitor-1 (PAI-1), is a key component of the plasma fibrinolytic system that regulates thrombolysis and has been implicated in pathological conditions including fibrosis and inflammation (Miyata, 2025; Zhou et al., 2025). In our study, we also found that HH exposure upregulated the expressions of *Kdr*, *Eng*, *Vegfa*, *Acta2*, *Vwf*, and *Serpine1*, which likely contributed to the BBB disruption and neuroinflammation.

The BBB is pivotal in preserving brain homeostasis and neurological function. In this study, HH-exposed mice exhibited significantly increased EB leakage and decreased expression of the tight junction proteins ZO-1 and Occludin. Given that tight junction proteins are essential for maintaining BBB function, their downregulation provides a mechanistic basis for the observed hyperpermeability. These findings align with the established link between BBB disruption and cognitive impairment (Che et al., 2024), suggesting that HH-induced BBB damage may contribute to the memory deficits observed in our model.

KEGG pathway enrichment analysis indicated that the PI3K/AKT signaling pathway was the most significantly enriched in DEGs. The PI3K/AKT pathway is a key modulator of cellular processes such as survival, apoptosis, and cognitive function in the central nervous system (Wang et al., 2022). PI3K activates AKT by promoting the production of phosphatidylinositol 3,4,5-trisphosphate (PIP3) (Kearney et al., 2021). AKT, in turn, regulates downstream signaling molecules, including NF-κB and Nrf2. AKT negatively regulates NF-κB to suppress pro-inflammatory cytokine expression, while it positively regulates Nrf2 to improve cellular resistance to oxidative stress (Bayo Jimenez et al., 2022; Fu et al., 2022). Inhibition of the PI3K/AKT signaling pathway has been linked to impaired learning and memory induced by different stress (Wang et al., 2021; Yang et al., 2023a; Xiong et al., 2024). In addition, PI3K/AKT signaling pathway is also participated in the pathogenesis of HH induced injury, such as cerebral edema (Gong et al., 2018), lung injury (Li et al., 2023), myocardial injury (Wang et al., 2020), and pulmonary hypertension (Ji et al., 2022). Combined with transcriptome data and previous literature, it is speculated that PI3K/AKT signaling pathway has a pivotal role in the occurrence and development of HACD.

To elucidate the function of PI3K/AKT signaling pathway in HACD, we first validated four hub genes associated with this pathway-*Spp1, Vwf, Vegfa, and Kdr*-using qRT-PCR. The trends of these genes were consistent with our RNA-seq results, confirming their involvement in HACD. Given the crucial role of these genes, we next sought to determine whether the PI3K/AKT pathway itself is functionally altered following HH exposure. We detected the phosphorylation status of PI3K and AKT, as only their phosphorylated forms are enzymatically active (Chu et al., 2018). Western blot analysis revealed that there were significant decreases in the p-PI3K/PI3K and p-AKT/AKT ratios in the hippocampal tissue of mice suffering from HACD compared to the Con group, suggesting that the PI3K/AKT signaling pathway was inhibited. Furthermore, we examined the expression of downstream effectors. As expected, the expression levels of Nrf2 and HO-1 were significantly decreased following HH exposure, while the p-NF-κB/NF-κB ratio was elevated, indicating NF-κB pathway activation. These findings further suggest that the inhibition of the PI3K/AKT signaling pathway may be involved in HACD, potentially by promoting neuroinflammation (via NF-κB activation) and impairing antioxidant defense mechanisms (via Nrf2/HO-1 downregulation).

There were several limitations in this study. First, due to the observational design, causal conclusions regarding PI3K/AKT inhibition in HACD cannot be drawn; further studies using pathway activators are needed. Second, the limited sample size may have restricted our capacity to identify significant alterations in key PI3K/AKT pathway genes, specifically PIK3CA and AKT1. Third, we did not explore other PI3K/AKT-related processes such as synaptic neuroplasticity and apoptosis. Fourth, we used male mice that are more sensitive to the plateau environment in our study, but this can lead to gender bias. Fifth, our analysis focused on the whole hippocampus rather than each subregion of the hippocampus, which may exhibit distinct responses to HH stimulation. Future studies should address these aspects.

To sum up, our study provided evidence that HH exposure is associated with cognitive dysfunction, accompanied by inhibition of the PI3K/AKT signaling pathway, increased oxidative stress, neuroinflammation, and BBB disruption in the hippocampus. These findings suggest that the PI3K/AKT pathway may play a crucial role in the pathophysiology of HACD and could represent a potential therapeutic target for further investigation.

## Data Availability

The datasets presented in this study can be found in online repositories. The names of the repository/repositories and accession number(s) can be found below: RNA-Seq data can be found online at: https://www.ncbi.nlm.nih.gov/geo/query/acc.cgi?acc=GSE290548.
