# Supplementary material for: Transcriptomic analysis and experimental verification reveal the involvement of PI3K/AKT signaling pathway in high-altitude cognitive dysfunction
Source: Front Physiol. 2026 Apr 23;17:1781613. doi: 10.3389/fphys.2026.1781613 (PMC13149069; doi:10.3389/fphys.2026.1781613)
Supplement: Supplementary file 1 [file DataSheet1.docx]

**Supplementary Information**

**Transcriptomic analysis and experimental verification reveal the involvement of PI3K/AKT signaling pathway in high-altitude cognitive dysfunction**

Yu Xin^1,2^, Chenyu Yang^2^, Gege Wang^1,2^, Huiping Ma^2*^, Linlin Jing^1,2*^

^1^Department of Pharmacy, The First Affiliated Hospital of Xi'an Jiaotong University, Xi'an, Shaanxi 710061, China.

^2^Department of Pharmacy, The 940th Hospital of Joint Logistics Support force of PLA, Lanzhou, Gansu, 730050, China.

**Correspondence**

**Linlin Jing,** Department of Pharmacy, The First Affiliated Hospital of Xi'an Jiaotong University, NO.277 Yanta West Road, Yanta District, Xi’an, Shaanxi, 710061, People’s Republic of China.

E-mail address: [jinglinlin@xjtufh.edu.cn](mailto:jinglinlin@xjtufh.edu.cn)

**Huiping Ma**, Department of pharmacy, the 940th Hospital of Joint Logistics Support force of PLA, NO.333 Binhe South Road, Qilihe District, Lanzhou, 730050, Gansu, People’s Republic of China*.*

E-mail addresses: [huipingmacyk@163.com](mailto:huipingmacyk@163.com)

Table S1 Sample sequencing data quality summary

| **sample** | **library** | **raw_reads** | **raw_bases** | **clean_reads** | **clean_bases** | **error_rate** | **Q20** | **Q30** | **GC_pct** |
| --- | --- | --- | --- | --- | --- | --- | --- | --- | --- |
| **Mod-1** | **FRAS230110328-2r** | **39506620** | **5.93G** | **37331544** | **5.6G** | **0.03** | **97.56** | **93.63** | **48.67** |
| **Mod-2** | **FRAS230110330-2r** | **39417106** | **5.91G** | **39270204** | **5.89G** | **0.03** | **97.25** | **93.33** | **50.31** |
| **Mod-3** | **FRAS230110331-2r** | **43745780** | **6.56G** | **40081542** | **6.01G** | **0.03** | **97.37** | **93.34** | **48.36** |
| **Mod-4** | **FRAS230110332-2r** | **43307774** | **6.5G** | **40918732** | **6.14G** | **0.03** | **97.59** | **93.74** | **48.85** |
| **Con-1** | **FRAS230110335-1r** | **43213992** | **6.48G** | **40571858** | **6.09G** | **0.03** | **97.54** | **93.76** | **47.96** |
| **Con-2** | **FRAS230110336-1r** | **42699498** | **6.4G** | **40517400** | **6.08G** | **0.03** | **97.67** | **93.92** | **48.2** |
| **Con-3** | **FRAS230110337-1r** | **41494616** | **6.22G** | **38837198** | **5.83G** | **0.03** | **97.48** | **93.58** | **49.31** |
| **Con-4** | **FRAS230110338-1r** | **44335194** | **6.65G** | **41757212** | **6.26G** | **0.03** | **97.67** | **93.86** | **48** |

Table S2 Statistics of comparison between samples and reference genomes

| **sample** | **total_reads** | **total_map** | **unique_map** | **multi_map** | **read1_map** | **read2_map** | **positive_map** | **negative_map** | **splice_map** | **unsplice_map** | **proper_map** |
| --- | --- | --- | --- | --- | --- | --- | --- | --- | --- | --- | --- |
| **Mod-1** | **37331544** | **35369572(94.74%)** | **33504444(89.75%)** | **1865128(5.0%)** | **16742812(44.85%)** | **16761632(44.9%)** | **16743757(44.85%)** | **16760687(44.9%)** | **8292120(22.21%)** | **25212324(67.54%)** | **31855732(85.33%)** |
| **Mod-2** | **39270204** | **36268764(92.36%)** | **34920970(88.92%)** | **1347794(3.43%)** | **17442959(44.42%)** | **17478011(44.51%)** | **17473279(44.5%)** | **17447691(44.43%)** | **9306456(23.7%)** | **25614514(65.23%)** | **33051736(84.16%)** |
| **Mod-3** | **40081542** | **37663284(93.97%)** | **35565253(88.73%)** | **2098031(5.23%)** | **17789084(44.38%)** | **17776169(44.35%)** | **17774323(44.35%)** | **17790930(44.39%)** | **7992072(19.94%)** | **27573181(68.79%)** | **33528188(83.65%)** |
| **Mod-4** | **40918732** | **38627247(94.4%)** | **36631085(89.52%)** | **1996162(4.88%)** | **18344525(44.83%)** | **18286560(44.69%)** | **18307981(44.74%)** | **18323104(44.78%)** | **8886444(21.72%)** | **27744641(67.8%)** | **34556854(84.45%)** |
| **Con-1** | **40571858** | **38491274(94.87%)** | **36062405(88.89%)** | **2428869(5.99%)** | **18053780(44.5%)** | **18008625(44.39%)** | **18023494(44.42%)** | **18038911(44.46%)** | **8182642(20.17%)** | **27879763(68.72%)** | **33981128(83.76%)** |
| **Con-2** | **40517400** | **38702715(95.52%)** | **36623512(90.39%)** | **2079203(5.13%)** | **18311625(45.19%)** | **18311887(45.2%)** | **18304828(45.18%)** | **18318684(45.21%)** | **8958421(22.11%)** | **27665091(68.28%)** | **34802818(85.9%)** |
| **Con-3** | **38837198** | **36359760(93.62%)** | **34823986(89.67%)** | **1535774(3.95%)** | **17412190(44.83%)** | **17411796(44.83%)** | **17412387(44.83%)** | **17411599(44.83%)** | **8276658(21.31%)** | **26547328(68.36%)** | **32812358(84.49%)** |
| **Con-4** | **41757212** | **39359477(94.26%)** | **37114613(88.88%)** | **2244864(5.38%)** | **18585982(44.51%)** | **18528631(44.37%)** | **18543723(44.41%)** | **18570890(44.47%)** | **9808098(23.49%)** | **27306515(65.39%)** | **34884914(83.54%)** |


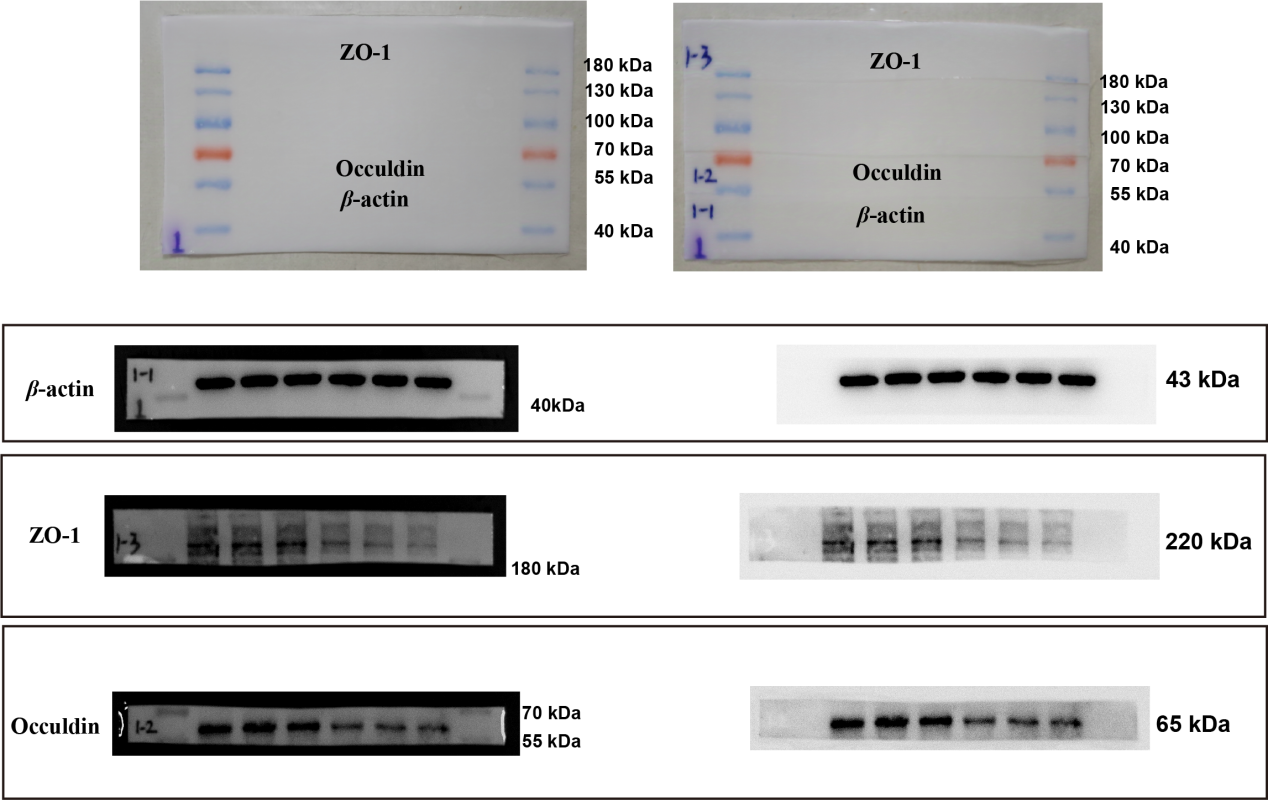


Figure S1. Uncropped western blots used for Figure 4B.


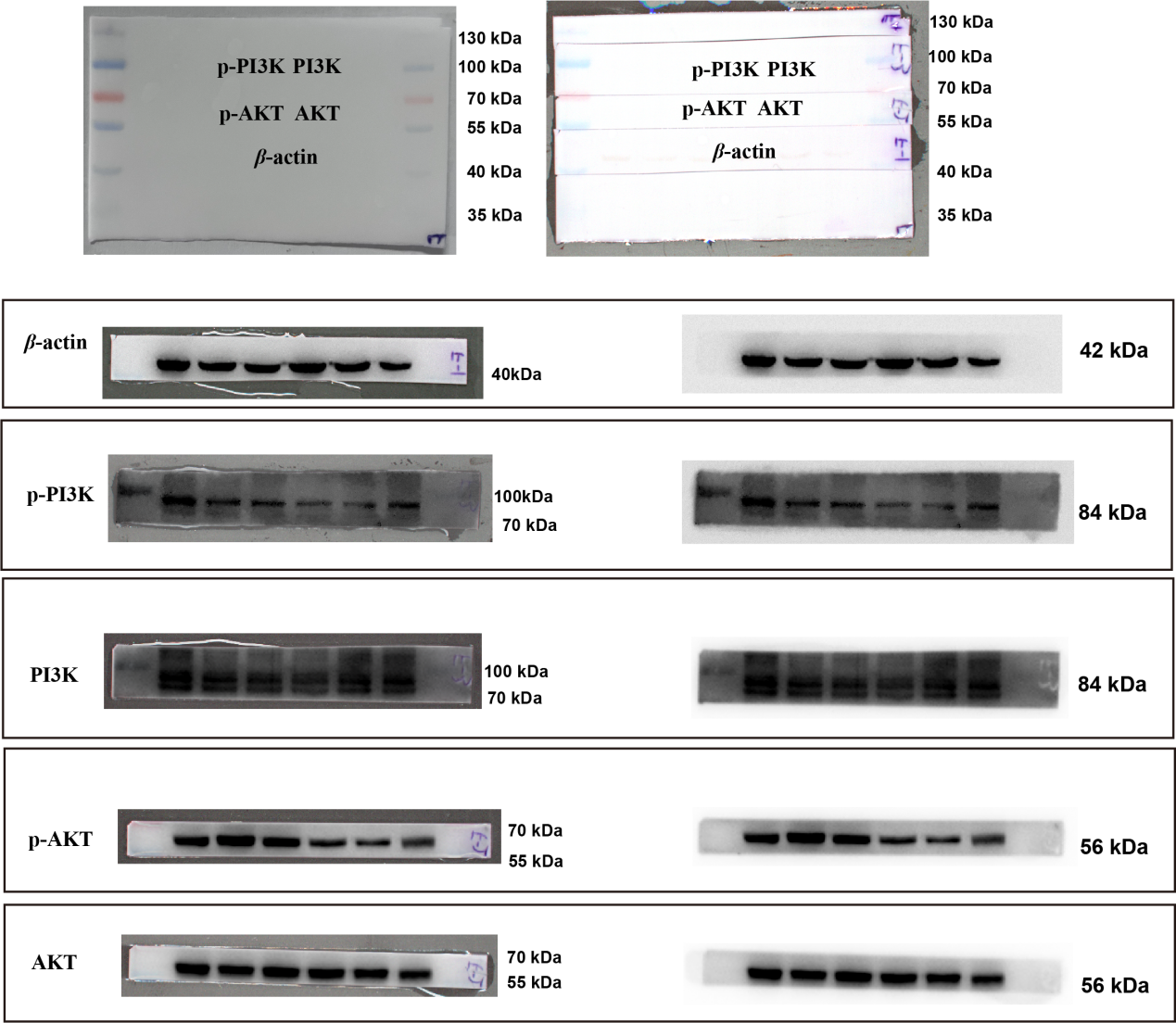


Figure S2. Uncropped western blots used for Figure 8B.


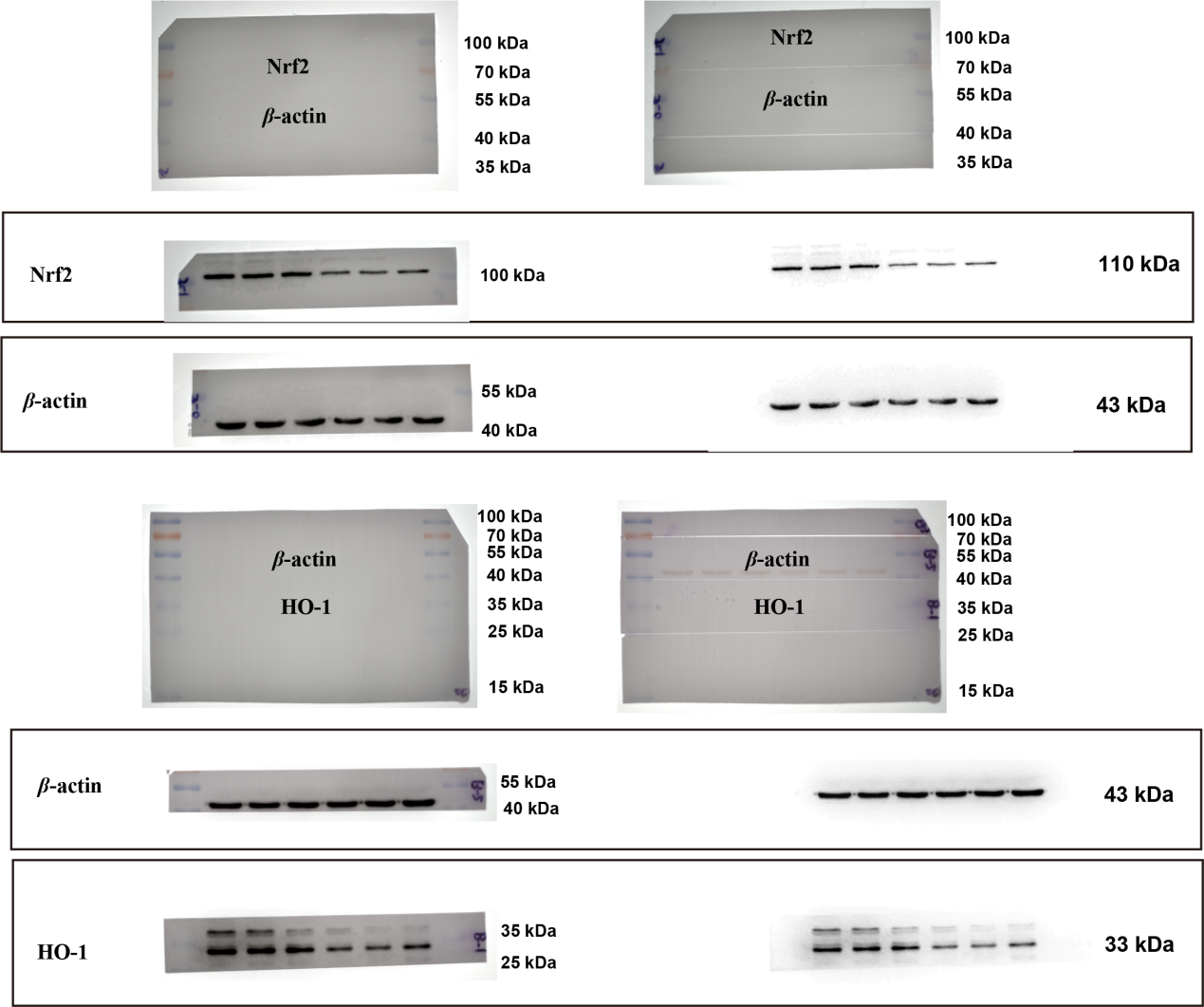


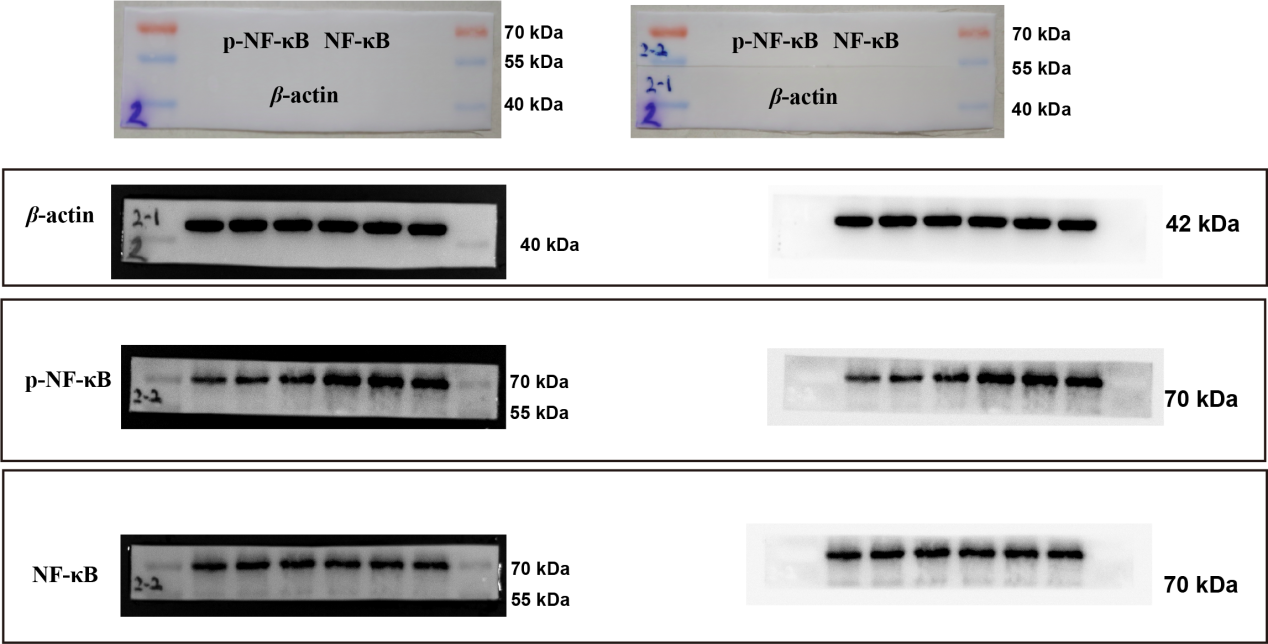


Figure S3. Uncropped western blots used for Figure 8E.
